# MicroRNA-155 as a proinflammatory regulator via SHIP-1 down-regulation in acute gouty arthritis

**DOI:** 10.1186/ar4531

**Published:** 2014-04-07

**Authors:** Hye Mi Jin, Tae-Jong Kim, Jung-Ho Choi, Moon-Ju Kim, Young-Nan Cho, Kwang-Il Nam, Seung-Jung Kee, Jang Bae Moon, Seok-Yong Choi, Dong-Jin Park, Shin-Seok Lee, Yong-Wook Park

**Affiliations:** 1 Department of Rheumatology, Research Institute of Medical Sciences, Chonnam National University Medical School and Hospital, 671 Jebongro, Dong-gu, Gwangju 501-757, Republic of Korea; 2 Department of Anatomy, Chonnam National University Medical School, 160 Baekseo-ro, Gwangju 501-840, South Korea; 3 Department of Laboratory Medicine, Chonnam National University Medical School and Hospital, 42, Jebong-ro, Gwangju 501-757, South Korea; 4 National Research Laboratory for Regulation of Bone Metabolism and Disease, Medical Research Center for Gene Regulation, Chonnam National University Medical School, 42, Jebong-ro, Gwangju 501-757, South Korea; 5 Department of Biomedical Sciences, Chonnam National University Medical School, Gwangju, Republic of Korea

## Abstract

**Introduction:**

Gout is characterized by episodes of intense joint inflammation in response to intra-articular monosodium urate monohydrate (MSU) crystals. miR-155 is crucial for the proinflammatory activation of human myeloid cells and antigen-driven inflammatory arthritis. The functional role of miR-155 in acute gouty arthritis has not been defined. Therefore, the aim of this study was to examine the role of miR-155 in pathogenesis of acute gouty arthritis.

**Methods:**

Samples from 14 patients with acute gouty arthritis and 10 healthy controls (HCs) were obtained. Peripheral blood mononuclear cells (PBMCs) and synovial fluid mononuclear cells (SFMCs) were cultured *in vitro* with MSU crystals, and gene expression (human miR-155 and SHIP-1) were assessed by real-time PCR. THP-1 cells were stimulated by MSU crystals and/or miR-155 transfection and then subjected to Western blot analysis. Levels of human tumor necrosis factor-alpha (TNF-α) and interleukin (IL)-1β in cell culture supernatants were measured by Luminex. Immunohistochemistry was performed on formalin-fixed gout tissues with anti–SHIP-1 antibody. A C57BL/6 J male mouse model of gout was used to analyze the expressions of miR-155, SHIP-1, and inflammatory cytokines.

**Results:**

The samples from gouty arthritis were highly enriched in miR-155, with levels of expression being higher than those found in PBMC from HC. Treatment of the cells with MSU crystals strongly induced miR-155. In addition, overexpression of miR-155 in the cells decreased levels of SHIP-1 and promoted production of MSU-induced proinflammatory cytokines, such as TNF-α and IL-1β. Consistent with *in vitro* observations, miR-155 expression was elevated in the mouse model of gout. The production of inflammatory cytokines was markedly increased in MSU crystal induced peritonitis mice.

**Conclusions:**

Overexpression of miR-155 in the gouty SFMC leads to suppress SHIP-1 levels and enhance proinflammatory cytokines.

## Introduction

Gout is characterized by episodes of intense joint inflammation, in response tointra-articular monosodium urate monohydrate (MSU) crystals [1]. This inflammatory response is specifically initiated by inflammasome activation within monocytes/macrophages, causing activation of inflammatory cytokines within the joint [2].

miRNA are noncoding RNA oligonucleotides that have been highly conserved during evolution and which have recently emerged as potent regulators of gene expression of posttranscriptional regulators [3]. miRNA have been identified that appear to be critical for fine-tuning many biological processes, and offers the prospect of multiple targets [4,5]. Emerging data suggest that single miRNA species can profoundly alter the phenotype and outcome of immune responses [6-8].

miR-155 is crucial for the proinflammatory activation of human myeloid cells and antigen-driven inflammatory arthritis [9]. Peripheral blood mononuclear cells (PBMC) in rheumatoid arthritis (RA) express elevated levels of miR-155, which is particularly associated with disease activity [10]. Also, miR-155 is up-regulated in the synovial membrane of RA [11]. miR-155 has a dual effect on the control of autoimmune-triggered destructive arthritis. miR-155 deficiency inhibits the generation of pathogenic self-reactive T and B cell responses. On the other hand, miR-155 controls the development of local bone destruction [12]. The functional role of miR-155 in gouty arthritis has not been defined. Therefore, the aim of this study was to examine the role of miR-155 in the pathogenesis of acute gouty arthritis.

## Methods

### Sample collection

Samples from 14 patients with acute gouty arthritis and 10 healthy controls (HCs) were obtained. The clinical features of the subjects are summarized in Table 1. The paired samples of peripheral blood and synovial samples were obtained from patients with joint effusion. The presence of MSU crystals in all synovial fluid samples was confirmed by polarizing light microscopy. It would have been inappropriate to obtain synovial tissue samples from HCs for comparison with those obtained from patients with gouty arthritis. Hence, we used synovial tissue samples obtained from osteoarthritis (OA) patients, who underwent surgery of the knee joint and who did not have a medical history of inflammatory arthritis, as a representative sample of non-inflammatory synovium based on a previous report [9]. The study was approved by the Institutional Review Board of Chonnam National University Hospital, and written informed consent was obtained from all participants.

**Table 1:** Clinical and laboratory characteristics of patients with gouty arthritis and healthy controls

	**Healthy controls**	**Patient**
Total number	10	14
Age, mean (range) years*	51 (25 to 65)	55 (31 to 68)
Male, number (%)*	10 (100)	14 (100)
Duration of acute gout, mean (range) days	NA	2 (1 to 4)
Co-morbidity	NA	
Diabetes, number (%)		3 (21.4)
Hypertension, number (%)		4 (28.5)
Hypercholesterolemia, number (%)		1 (7.1)
Cardiovascular disease, number (%)		2 (14.3)
Current medicines	NA	
Naive, number (%)		14 (100)
Location of arthritis	NA	
First MTP joint, number (%)		10 (71.4)
Knee joint, number (%)		12 (72.3)
Ankle joint, number (%)		2 (14.3)
Serum urate level, mean (range) mg/dl	NA	7 (5 to 12)
ESR, mean (range, mm/h)	NA	55 (45 to 61)
CRP, mean (range, mg/dL)	NA	12.5 (5.8 to 18.3)

CRP, C-reactive protein; ESR, erythrocyte sedimentation rate; MTP, metatarsophalangeal; NA, not applicable. *Not significant.

### MSU crystal synthesis

MSU crystals were prepared by recrystallization from uric acid as previously described [13]. Briefly, 0.4 mg of uric acid was dissolved in 80 ml of boiling distilled water containing 2.45 ml of 1 N NaOH. After adjusting the pH of the solution to 7.2 with HCl, the solution was gradually cooled by stirring at room temperature and stored overnight at 4°C. The crystals that formed were sterilized by heating at 180°C for 2 h and suspended in PBS at a concentration of 1 mg/ml. The crystals obtained by this method were of comparable size (5 to 25 μm long) and needle-shaped, negatively birefringent crystals observed by compensated polarized light microscopy. Endotoxin levels in MSU crystal preparations, as assessed by Limulus amoebocyte cell lysate assay (Lonza, Walkersville, MD, USA), were less than 0.01 IU/ml.

### Isolation of PBMCs and synovial fluid mononuclear cells (SFMCs), and MSU stimulation

Peripheral venous blood and synovial fluid samples were collected in heparin-containing tubes. PBMCs and SFMCs were isolated by density-gradient centrifugation using Ficoll-Paque Plus solution (Amersham Biosciences, Uppsala, Sweden). Freshly isolated PBMCs were suspended in a complete medium, consisting of RPMI 1640, 2 mM L-glutamine, 100 units/ml of penicillin, and 100 μg/ml of streptomycin, supplemented with 10% FBS (Gibco BRL, Grand Island, NY, USA), and then plated on 6-cm dishes at a density of 5 × 10^5^ cells/ml. After MSU crystals were added directly to the culture plates, cells were cultured in completed media for the indicated times (4, 12, 24, and 48 h) at 37°C in a 5% CO_2_ humidified incubator for evaluation of miR-155 and *Src homology 2-containing inositol phosphatase (SHIP)-1* gene level.

### Real-time polymerase chain reaction (PCR)

Total RNA was isolated using the miRNeasy kit (Qiagen, Valencia, CA, USA). The miScript Reverse Transcription Kit (Qiagen) was used for cDNA preparation. MiScript primer assay (Qiagen) was used for semiquantitative determination of the expression of human miR-155 (MS00003605). The primer sequences were as follows: for human *SHIP-1*, 5′-GCC-TAC-ACC-AAG-CAG-AAA-GC-3′ (forward), 5′-GGA-CCG-TTC-TTG-GAG-ACA-AA-3′ (reverse). The expression of *U6B* snRNA or *β-actin* was used as an endogenous control. A threshold cycle (CT) was observed in the exponential phase of amplification, and quantification of relative expression levels was performed using standard curves for target genes and the endogenous control. Geometric means were used to calculate the ΔΔCT (delta-delta CT) values and are expressed as 2-ΔΔCT. The value of each control sample was set at 1 and was used to calculate the fold-change of the target genes.

### Cell culture and stimulation

Freshly isolated PBMCs were cultured in RPMI 1640 containing 10% FBS with macrophage colony-stimulating factor (M-CSF; 30 ng/ml; PeproTech, London, UK). After 7 days, adherent cells were used as macrophages [14]. The acute human monocytic leukaemia THP-1 cell line (TIB-202; American Type Culture Collection, Manassas, VA, USA) was used in a well-characterized model of acute gout [2]. THP-1 cells were seeded on 6-cm dishes at a density of 5 × 10^5^ cells/ml: 50nM miR-155 (UUAAUGCUAAUCGUGAUAGGGGU) or 50 nM scramble control (CCUACGCCACCAAUUUCGU) was mixed with Lipofectamine 2000 reagent (Invitrogen, Carlsbad, CA, USA) in serum-free RPMI and transfected into the cells per the manufacturer’s protocol. Optimal miR-155 expression was confirmed at 24 h post-transfection by the qPCR analysis (See Additional file 1). After transfection, cells were treated with phorbol 12-myristate 13-acetate/ionomycin (PMA; 100 ng/ml) for 3 h to detect the levels of inflammatory cytokines. This treatment increases the phagocytic properties of the cells and induces a constitutive production of proinflammatory cytokines [2]. After PMA stimulation, cells were washed and then stimulated with MSU (100 ug/ml) for 24 h, as described [15]. Human TNF-α and IL-1β levels in cell culture supernatants were measured using Luminex (Millipore, Billerica, MA, USA) according to the instructions of the manufacturer.

### Western blot analysis

Cells were harvested after washing with ice-cold PBS and then lysed in extraction buffer (50 mM Tris/HCl (pH 7.5), 150 mM NaCl, 2 mM EDTA, 1% Triton X-100, 1% sodium deoxycholate, 0.1% sodium dodecyl sulfate (SDS) and 0.01% protease inhibitor cocktail). Whole-cell lysates were subjected to 10% SDS- PAGE and western blot analysis. Primary antibodies included anti-actin (Sigma-Aldrich, St Louis, MO, USA), anti-SHIP-1, anti-phospho-Akt, anti-Akt and anti-IκB-α (Cell Signaling Technology, Beverly, MA, USA) antibodies, and horseradish peroxidase (HRP)-conjugated secondary antibodies (Amersham Biosciences, Pittsburgh, PA, USA) were used and blots were developed with enhanced chemiluminescence (ECL) solution (Millipore Corporation, Billerica, MA, USA). Signals were detected and analyzed using an LAS3000 luminescent image analyzer (Fuji Photo Film, Tokyo, Japan) (See Additional file 2).

### Immunohistochemical stain

The gout and osteoarthritis specimens were fixed in 4% paraformaldehyde. The paraffin sections were immunohistochemically stained using LSAB2 system-HRP kit (DakoCytomation, Carpinteria, CA, USA), according to the manufacturer’s instruction. In brief, the sections were deparaffinized, hydrated, and placed in peroxidase block solution for 10 minute. After washing three times in PBS, the sections were incubated with monoclonal mouse SHIP-1 antibody (1:400, Santa Cruz Biotechnology, Santa Cruz, CA, USA) for 2 h, treated with biotinylated link solution for 20 minutes, and streptavidin-HRP solution applied for 20 minutes. For the negative control, primary antiserum was replaced with PBS. Consequently the sections were treated with 3,3-diaminobenzidine (DAB) substrate-chromogen solution for 10 minutes and counterstained with Harris hematoxylin. Images were photographed on a light microscope (Leica, Wetzlar, Germany).

### *In vivo* mouse peritonitis model

Male C57BL/6 J mice used for the experiments were aged 8 to 10 weeks. Peritonitis was induced by intra-peritoneal injection of 3 mg MSU crystals in 250 μl PBS [16]. After 24 h, mice were killed by CO_2_ exposure and peritoneal cavities were washed with 4 ml of PBS containing 25 units/ml heparin. The cells were collected by peritoneal lavage and analyzed for the expressions of miR-155 (MS00001701), inflammatory cytokines by real-time PCR, and anti-mouse SHIP-1 (Santa Cruz Biotechnology) antibody was used for western blot. The sequences of the primers used were as follows: for *glyceraldehydes-3-phosphate dehydrogenase(GAPDH)*, 5′-TGA-CCA-CAG-TCC-ATG-CCA-TCA-CTG-3′ (forward), 5′-CAG-GAG-ACA-ACC-TGG-TCC-TCA-GTG-3′ (reverse); for *IL-1β*, 5′- CTC-GTG-CTG-TCG-GAC-CCA-TAT-GAG-3′ (forward), 5′-TGT-ACC-AGT-TGG-GGA-ACT-CTG-CAG-3′ (reverse); for *TNF-α*, 5′-GGC-TCC-AGG-CGG-TGC-TTG-3′ (forward), 5′- GGG-CTA-CAG-GCT-TGT-CAC-TCG -3′ (reverse).

### Statistical analyses

The Mann–Whitney *U*-test was performed for continuous variables that were not normally distributed, and chi-square tests were used for categorical variables. The Kruskal-Wallis test was used for the comparison of cytokines among groups. Dunn’s test was used post-hoc to compare all pairs of groups. A *P-*value <0.05 was considered statistically significant. All statistical analyses were performed using SPSS version 17.0 software (SPSS, Chicago, IL, USA).

## Results

### Increased expression levels of miR-155 in SFMCs of gouty arthritis patients

**Figure 1:**
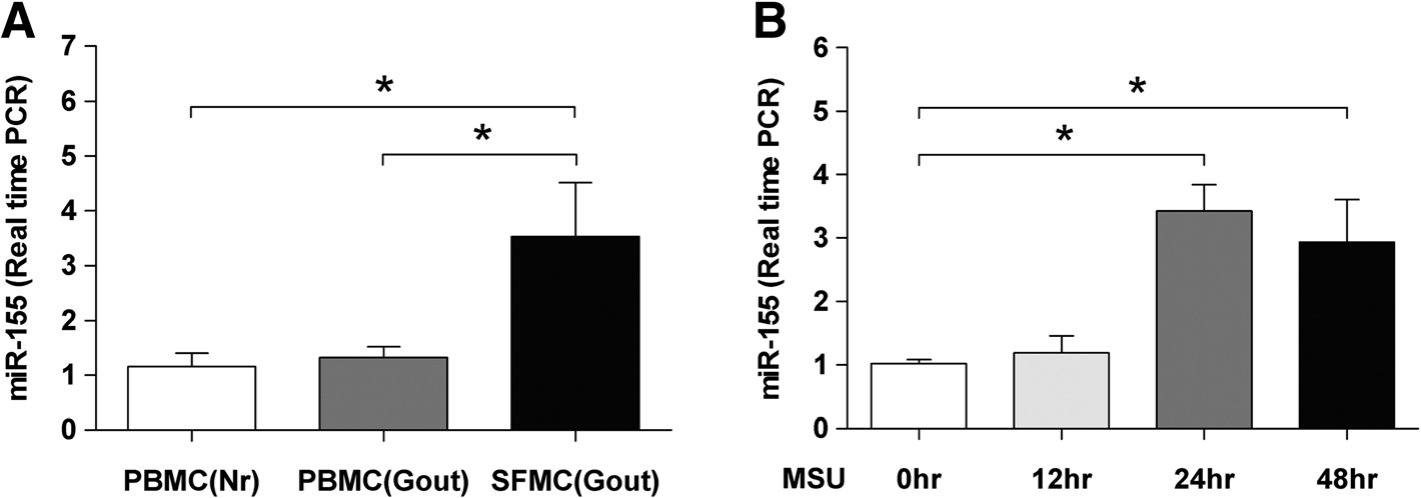
**miR-155 is overexpressed in synovial fluid mononuclear cells (SFMCs) from patients with gouty arthritis and up-regulated by stimulation with monosodium urate (MSU) crystals. (A)** Samples from 10 healthy controls (HCs), and paired ones of peripheral blood and synovial fluid from 14 patients with acute gouty arthritis were obtained. **(B)** Freshly isolated peripheral blood mononuclear cells (PBMCs) from HCs were cultured for the indicated times in the presence of MSU crystals (100 μg/ml). Total RNA was collected from each time point. Real-time PCR analysis was performed to determine the expressions of miR-155. Results are representative of nine independent experiments. Values are shown as the mean ± standard error of the mean (SEM). *p <0.05, Mann–Whitney *U*-test.

We performed expression analysis of miR-155 in PBMCs and SFMCs. The expression levels of miR-155 in SFMCs from gout patients were significantly higher than those in PBMC from HCs and patients (*P* <0.05) (Figure 1A). Given our observation that miR-155 was over-expressed in SFMCs of gout patients, we investigated whether MSU, which is the key factor in gout pathogenesis, could stimulate miR-155 expression. Freshly isolated PBMCs from HCs were stimulated with MSU crystals for the indicated times, and the expression level of miR-155 was determined by real-time PCR. Expression of miR-155 was strongly induced by stimulation of MSU crystals after 24 h (Figure 1B).

### Decreased expression levels of SHIP-1 in SFMCs of gouty arthritis patients

**Figure 2:**
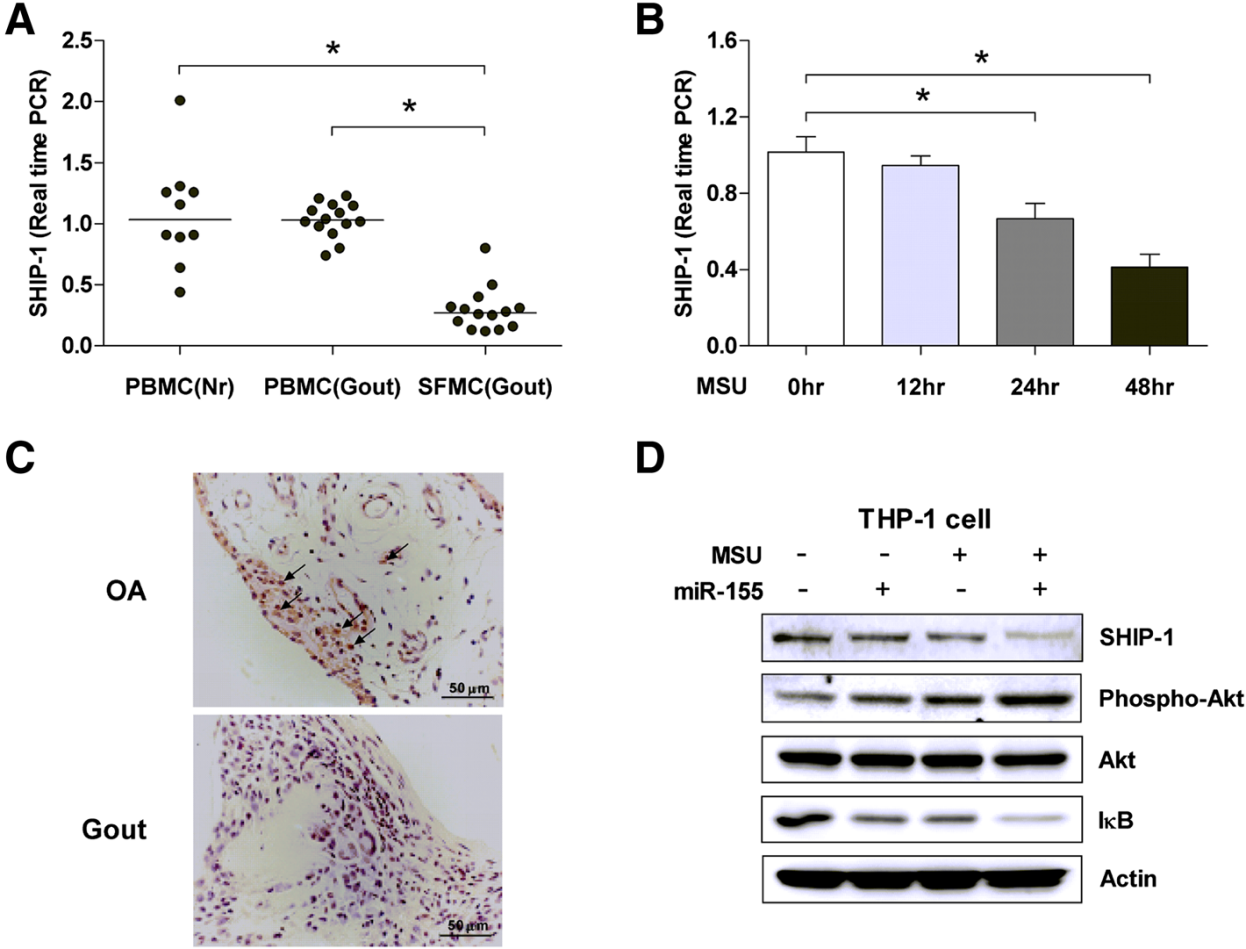
**Decreased expression of Src homology 2-containing inositol phosphatase-1 (SHIP-1) in gouty arthritis. (A)** Expression of mRNA for *SHIP-1* in peripheral blood mononuclear cells (PBMCs) from healthy controls (HCs), PBMCs and synovial fluid mononuclear cells (SFMCs) of gouty arthritis patients. **(B)** PBMCs from HCs were cultured for the indicated times in the presence of the monosodium urate (MSU) crystals. Real-time PCR was performed to determine the mRNA expression of *SHIP-1*. Results are representative of three independent experiments. Values are shown as the mean ± standard error of the mean. **(C)** Synovial specimens from acute gouty arthritis and osteoarthritis patients were processed for immunohistochemical staining using anti-human SHIP-1 antibody. Scale bars in d = 50 μm. **(D)** THP-1 cells were stimulated or not, with MSU crystal and/or miR-155 tranfection. The scrambled controls were transfected in all of miR-155 negative cells to rule out the possibility of a non-specific anti-RNA response. It was analyzed by western blot for SHIP-1, phophrylated Akt, total Akt, IkB proteins. *P <0.05, Mann–Whitney *U*-test. OA, osteoarthritis.

As *SHIP-1* is a target of miR-155 [17-19], we focused on the expression of SHIP-1 in gouty SFMCs. The expression of *SHIP-1* mRNA was markedly down-regulated in SFMCs of gouty arthritis patients compared with that in PBMCs of gouty arthritis patients and normal controls (Figure 2A). Moreover, the level of *SHIP-1* mRNA was gradually decreased in PBMCs of healthy donors that were stimulated with MSU crystals (Figure 2B). We also compared the expression of SHIP-1 in the synovial tissue of gouty arthritis patients with that in the synovial tissue of osteoarthritis patients. Immunohistochemical staining revealed that the SHIP-1 protein was rarely expressed in synovial lining cells of gouty arthritis patients as compared to that in synovial lining cells of OA patients (Figure 2C). THP-1 cells were stimulated or not, with miR-155 and/or MSU crystal, as described in the Methods section. A representative immunoblot showed that the SHIP-1 protein level was partially decreased in the presence of miR-155 and/or MSU crystal stimulation; and more so, when the two conditions were simultaneously met. The phosphorylation of Akt was enhanced, when the levels of SHIP-1 were lowered. The level of NFκB inhibitor (IκB) was decreased under the same conditions, a probable consequence of the increased phosphorylation of Akt (Figure 2D). Given the interaction of miR-155 and SHIP-1, these data indicate that the SHIP-1 expression was suppressed in acute gouty arthritis due to the over expression of miR-155 and stimulation with MSU crystals.

### MiR-155 promotes the production of pro-inflammatory cytokines

**Figure 3:**
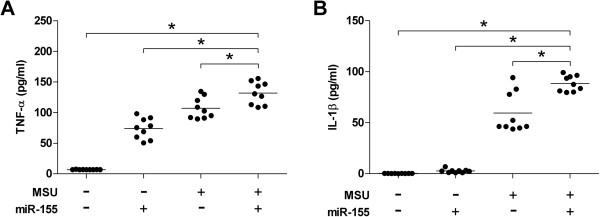
**miR-155 promotes the production of inflammatory cytokines over monosodium urate (MSU) crystal stimulation.** miR-155 or scrambled control were mixed with Lipofectamine 2000 Reagent in serum-free RPMI and transfected into the THP-1 cells. After 24 h of transfection, cells were treated with phorbol 12-myristate 13-acetate/ionomycin (PMA) (100 ng/ml) for 3 h. After stimulation with PMA, cells were washed and then stimulated with MSU (100 ug/ml) for 24 h. Human TNF-α **(A)** and IL-1β **(B)** levels in cell culture supernatants were measured using Luminex. *P <0.05, post-hoc analysis after the Kruskal-Wallis test.

Cytokine levels in cell culture supernatants were measured. Cells that encounter MSU crystals express a broad array of inflammatory mediators that contribute to acute gouty inflammation [20]. TNF-α level (mean ± standard error of the mean (SEM)) was significantly higher in the miR-155-transfected or in MSU crystal-stimulated samples than in controls (73.95 ± 5.65 versus 6.86 ± 0.09, and 107.11 ± 5.77 versus 6.86 ± 0.09, respectively). Furthermore, the TNF-α level was dramatically increased by both miR-155 transfection and MSU crystal stimulation (131.95 ± 6.14) (Figure 3A). The IL-1β level was not significantly higher in miR-155-only transfected supernatants than in controls (2.65 ± 0.59 versus 0.28 ± 0.03). However, the IL-1β level was increased by stimulation with MSU crystals only (59.42 ± 6.59). Even though miR-155 did not enhance the production of IL-1β, miR-155 promoted IL-1β levels adding to MSU crystal (88.45 ± 2.56) (Figure 3B). Thus, overexpression of miR-155 triggered the production of proinflammatory cytokines, which are strongly implicated in the pathogenesis of gouty arthritis.

### Contribution of miR-155 expression in the mouse model of gout

**Figure 4:**
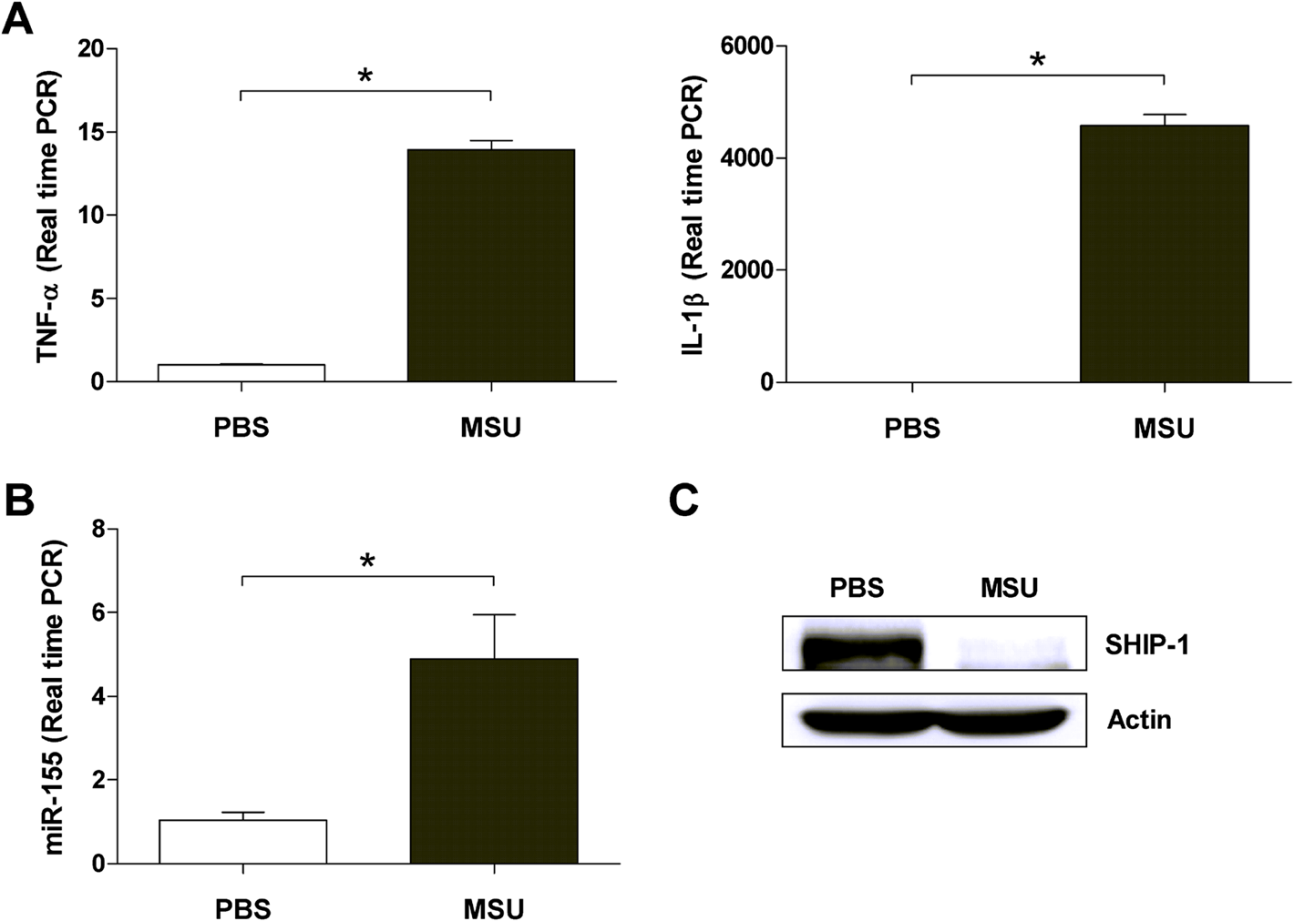
**Involvement of miR-155 as ascertained in the mouse gout peritonitis model.** Male C57BL/6 J mice (n = 3 per group) used for the experiments were aged 8 to 10 weeks. Peritonitis was induced by intraperitoneal injection of 3 mg monosodium urate crystals in 250 μl PBS. After 24 h, mice were killed and 4 ml PBS containing 25 units/ml heparin was injected into each peritoneal cavity. The peritoneal lavage fluid was aspirated and infiltrated cells were harvested. **(A)** Inflammatory cytokines were up-regulated. Real-time PCR was performed to determine the mRNA expression of TNF-α and IL-1β. **(B)** miR-155 is up-regulated in the gout mouse model. **(C)** Src homology 2-containing inositol phosphatase-1 (SHIP-1) protein level was suppressed on western blot analysis. Values are shown as the mean ± standard error of the mean. *P <0.05, Mann–Whitney *U*-test.

We next examined the contribution of miR-155 to the development of disease in the gout mouse model. Consistent with our *in vitro* observations, cells isolated from mice with MSU crystal-induced peritonitis showed significantly increased mRNA levels of *TNF-α* and *IL-1β* (Figure 4A). miR-155 expression was elevated in the mouse model of gout, compared to controls (Figure 4B). In addition, SHIP-1 protein levels were markedly suppressed in cells isolated from peritoneal lavage (Figure 4C).

## Discussion

Emerging data suggest that single miRNA species can profoundly alter the phenotype and outcome of immune responses. Dysregulation of miRNA levels has been reported in a number of pathologic conditions [3]. More than 100 miRNAs are expressed by cells of the immune system, and they have the potential to broadly influence the molecular pathways that control the development and function of immune responses [4]. Of those, miR-155 has been implicated in the differentiation and activation of cells of both the adaptive and the innate immune systems [4]. Expression of miR-155 has been demonstrated in the context of several autoimmune diseases, including RA. Up-regulated expression of miR-155 in PBMC and synovial membrane cells of RA patients has been observed [10,11]. miR-155 can be induced in macrophages in response to both bacterial and viral-derived antigens that activate Toll-like receptors (TLRs) [21,22].

Gout is an autoinflammatory disorder associated with deposition of MSU crystals in joints and peri-articular tissues. Reported advances suggest that the innate immune system drives the gouty inflammatory response to MSU [23]. However, given the redundant and highly cell-specific effects mediated by miRNAs species, the precise functional implications of miR-155 expression in the pathogenesis of gouty arthritis have remained obscure.

In this study, miR-155 was up-regulated in SFMCs from patients with gouty arthritis. Furthermore, our *in vitro* experiments showed that MSU crystals induced increased levels of miR-155. miRNA controls gene expression by targeting mRNA and triggering either translation repression or RNA degradation [3]. Therefore, the prospect of targeting multiple pathways simultaneously is attractive to optimize the neutralization of complex effector immune pathways. Given the evidence for direct interaction between miR-155 and SHIP-1 [17-19], we explored SHIP-1 in the present study. This molecule also has been reported to be a target of miR-155 in the human RA monocyte [9]. In our data, lower expressions of SHIP-1 from gouty SFMC were observed, comparing to those from HC. *In vitro,* SHIP-1 level was gradually decreased by MSU crystal-mediated stimulation. Interestingly, our histological observation also revealed that the expressions of SHIP-1 in acute gout samples were fewer than those in OA samples. In addition, SHIP-1 proteins were markedly reduced according to enhancing miR-155 level in monocytes. Thus, our data demonstrate that overexpression of miR-155 contribute to down-regulation of SHIP-1 in gouty SFMC.

Cells that encounter MSU crystals express a broad array of inflammatory mediators that contribute to acute gouty inflammation [15]. The role of SHIP-1 is essential for the suppressor activity of macrophages and is a potent inhibitor of many inflammatory pathways [24,25]. Recently, it has been shown that monocytes and macrophages overexpressing miR-155 exhibit decreased SHIP-1 expression that may lead to increased production of pro-inflammatory cytokines [9]. It can be assumed that the miR-155/SHIP pathway might be responsible for causing excessive pro-inflammatory activation in gouty arthritis. In our cellular biochemical results, a trend (*P*-value not significant) towards increasing levels of phosphatidylinositol triphosphate (PtdIns-3,4,5-P3) was observed after miR-155 transfection and MSU crystal stimulation (see Additional file 3). Although it appeared that MSU crystal increased PtdIns-3,4,5-P3 levels, this effect was not significantly amplified in the presence of miR-155. The rapid rise and transient accumulation are the main characteristics of PtdIns-3,4,5-P3. Therefore, the possible reason was due to the difficulties of the measurements. Even though the accuracy and sensitivity of the assay for phosphoinositides have been improved, tissue extracts contain large quantities of lipids other than the phosphoinositides, which might be expected to interfere nonspecifically. Among the downstream molecules of PtdIns-3,4,5-P3, Akt is known to phosphorylate IκB kinase (IKK) thereby promoting activation of NF-kB, which is a key factor for inflammatory cytokines [26]. Therefore, in our study, miR-155 decreased the SHIP-1 level, the levels of phosphorylation of Akt were increased, and as a probable consequence, the NF-kB pathway was activated.

With respect to inflammatory cytokines, our *in vitro* experiments demonstrate that MSU crystals trigger the production of TNF-α in supernatants from stimulated monocytes. Furthermore, miR-155 promoted MSU crystal-induced TNF-α production. Although we could not confirm the increased level of IL-1β induced by miR-155 alone, miR-155 aided IL-1β production in supernatants from MSU crystal-stimulated monocytes. In particular, the elevated levels of pro-inflammatory cytokines, such as IL-1β and TNF-α, in the synovial fluid of patients with gout [27,28], may be associated with miR-155-mediated regulation of monocyte/macrophage responses to MSU crystals. Taken together, these findings indicate that the pro-inflammatory phenotype in acute gouty arthritis could be regulated by aberrant expression of miR-155. Our *in vivo* data demonstrated that increased miR-155 expression following stimulation with MSU stimulation plays a role in down-regulation of SHIP-1 protein. Consistent with a crucial role of miR-155 in the regulation of pro-inflammatory cytokine production *in vitro*, investigations from gout mice model showed that the pro-inflammatory cytokine levels were enhanced in accordance with decreasing levels of SHIP-1.

We have to consider whether miR-155 enhanced proinflammatory cytokines via other pathways, in addition to SHIP-1 signaling on monocytes. It was demonstrated that Akt was directly activated through miR-155–mediated suppression of the phosphatase protein phosphatase 2A catalytic subunit alpha [29]. Jiang *et al.* demonstrated that miR-155 expression was inversely correlated with the *Suppressor of cytokine signaling 1 (SOCS1)* gene, that normally functions as a negative feedback regulator of Janus-activated kinase (JAK)/signal transducer, and activator of transcription (STAT) signaling [30]. A recent report also revealed that miR-155 may contribute to experimental autoimmune encephalomyelitis, by promoting the development of inflammatory T cells [31]. Further research on both direct and indirect effects of miR-155 on inflammation by the MSU crystal stimulation are required.

In summary, this study is the first research to examine the role of mirRNA in acute gouty arthritis. miR-155 was up-regulated in SFMCs of patients with gouty arthritis. The pro-inflammatory phenotype in acute gouty arthritis could be regulated by aberrant expression of miR-155. These data provide a strong proof-of-concept for miR-155-based therapeutic approaches that could modulate inflammation in acute gout.

## Conclusion

The miR-155 was up-regulated in SFMCs from patients with acute gouty arthritis. The increased expression of miR-155 in SFMCs was associated with lower expression of SHIP-1, an inhibitor of inflammation. Overexpression of miR-155 in SFMC led to down-regulation of SHIP-1. Then Akt/NF-kB pathway was activated, and as a consequence, the production of pro-inflammatory cytokines was enhanced.

## Abbreviations

CRP: C-reactive protein; ESR: erythrocyte sedimentation rate; HC: healthy control; IKK: IκB kinase; IL: interleukin; IκB: Nuclear factor of kappa light chain enhancer of B-cells inhibitor; JAK: Janus-activated kinase; MMP-3: metalloproteinase-3; MSU: monosodium urate; MTP: metatarsophalangeal; NF-κB: Nuclear factor kappa light chain enhancer of activated B cells; PBMC: peripheral blood mononuclear cell; PtdIn: phosphatidylinositol; RA: rheumatoid arthritis; SEM: standard error of mean; SFMC: synovial fluid mononuclear cell; SHIP: Src homology 2-containing inositol phosphatase; SOCS: Suppressor of cytokine signaling; STAT: Signal transducer and activator of transcription; TLR: Toll-like receptors; TNF: tumor necrosis factor.

## Competing interests

The authors declare that they have no competing interests.

## Authors’ contributions

HMJ: data collection and analysis, critical revision of the manuscript and manuscript writing. TJK: conception and design, data collection and analysis, manuscript writing and final approval of the manuscript, critical revision and final approval of the manuscript, financial support. JHC: data collection, critical revision and final approval of the manuscript. MJK: data collection, critical revision and final approval of the manuscript. YNC: data collection, critical revision and final approval of the manuscript. KIN: data collection, manuscript writing and final approval of the manuscript. SJK: data analysis, critical revision and final approval of the manuscript. JBM: data collection, critical revision and final approval of the manuscript. SYC: conception, critical revision and final approval of the manuscript. DJP: data collection, critical revision and final approval of the manuscript. SSL: data collection, critical revision and final approval of the manuscript. YWP: design, data collection and analysis, manuscript writing and final approval of the manuscript, critical revision and final approval of the manuscript, financial support. All authors read and approved the final manuscript.

## Supplementary Material

Additional file 1 **Efficient transfection of cells with miR-155.** THP-1 cells were transfected with miR-155 or control. After 24 h, cells were analyzed by real-time PCR for miR-155 expression. Data are shown as the means ± standard error of the mean of three experiments. Click here for file

Additional file 2 Original blot data. Click here for file

Additional file 3 **Phosphatidylinositol triphosphate (PtdIns-3,4,5-P3) level with miR-155 and/or monosodium urate (MSU) crystal stimulation.** THP-1 cells were transfected with miR-155 or scamble control. After transfection, cells were treated with or without MSU crystal. Cells were analyzed according to phosphoinositide mass assays (Analytical Biochemistry 313 (2003) 234–245). Data are shown as the means ± standard error of the mean of three experiments. Click here for file
