# Supplementary material for: MicroRNA-155 as a proinflammatory regulator via SHIP-1 down-regulation in acute gouty arthritis
Source: Arthritis Res Ther. 2014 Apr 7;16(2):R88. doi: 10.1186/ar4531 (PMC4060367; doi:10.1186/ar4531)
Supplement: Additional file 2 — Original blot data. [file ar4531-S2.ppt]

## Slide 1
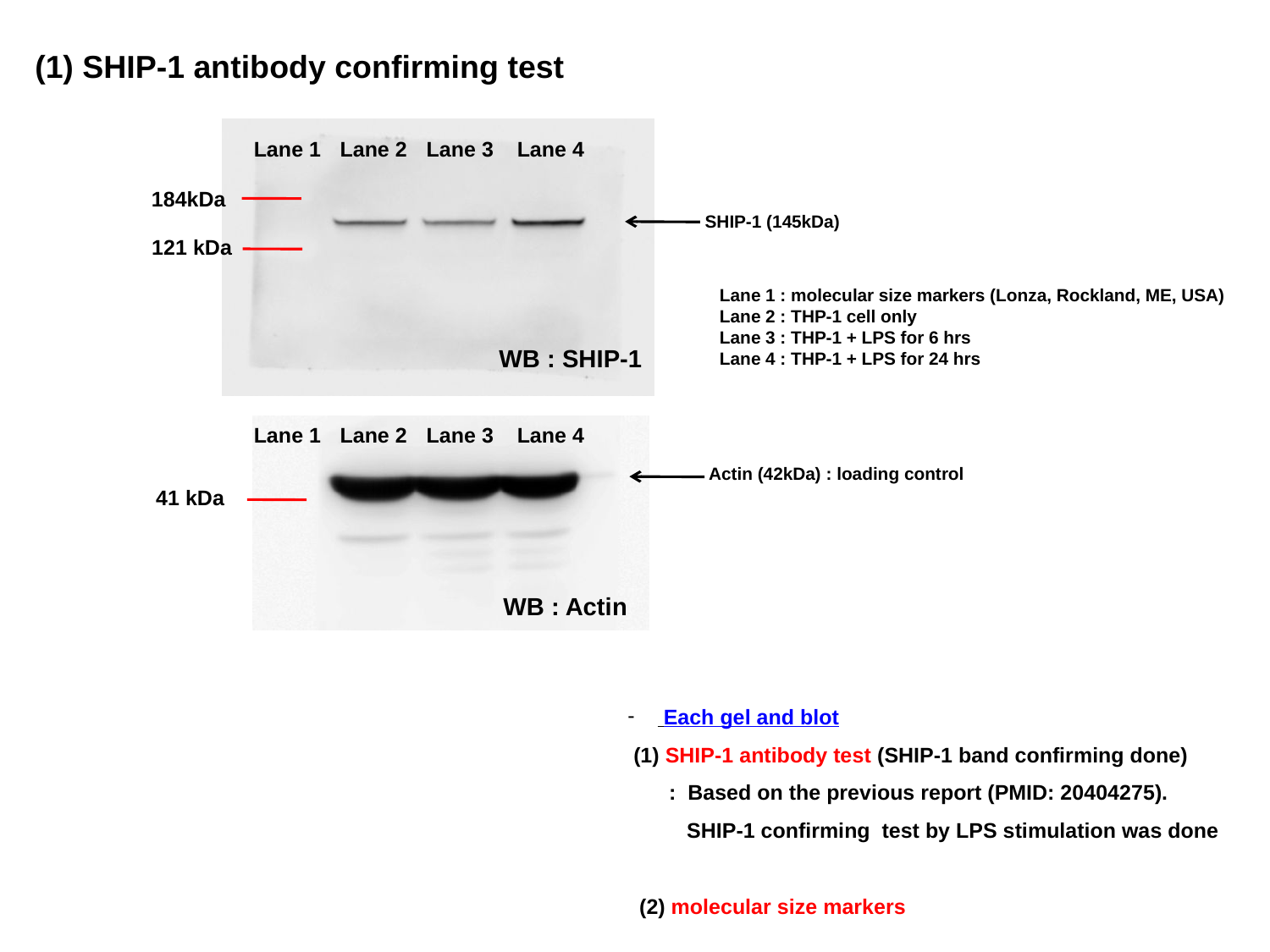

(1) SHIP-1 antibody confirming test
Lane 1
Lane 2
Lane 3
Lane 4
184kDa
SHIP-1 (145kDa)
121 kDa
Lane 1 : molecular size markers (Lonza, Rockland, ME, USA)
Lane 2 : THP-1 cell only
Lane 3 : THP-1 + LPS for 6 hrs
Lane 4 : THP-1 + LPS for 24 hrs
WB : SHIP-1
Lane 1
Lane 2
Lane 3
Lane 4
Actin (42kDa) : loading control
41 kDa
WB : Actin
 Each gel and blot
 (1) SHIP-1 antibody test (SHIP-1 band confirming done)
 : Based on the previous report (PMID: 20404275).
 SHIP-1 confirming test by LPS stimulation was done
 (2) molecular size markers

## Slide 2
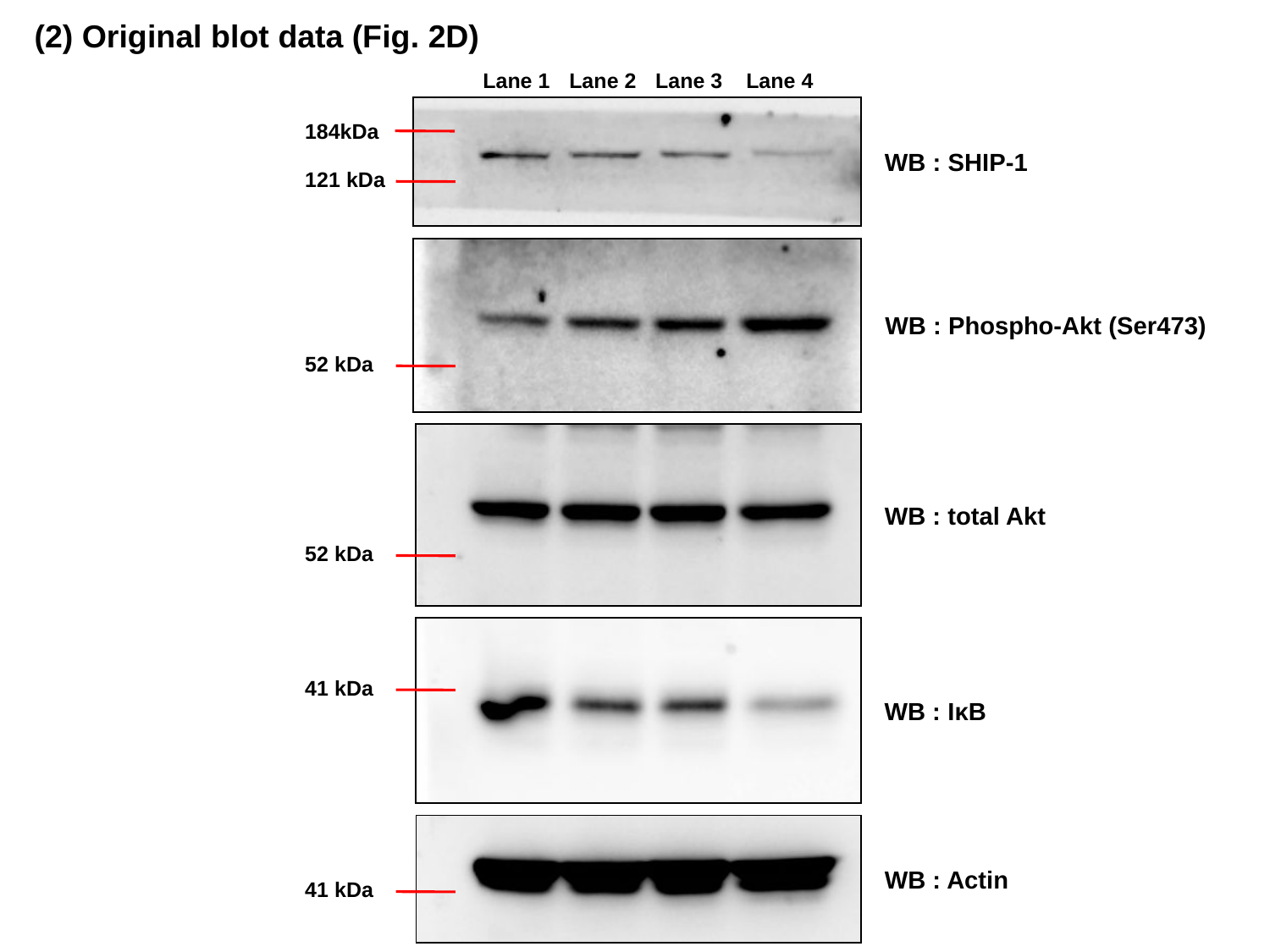

(2) Original blot data (Fig. 2D)
Lane 1
Lane 2
Lane 3
Lane 4
184kDa
WB : SHIP-1
121 kDa
WB : Phospho-Akt (Ser473)
52 kDa
WB : total Akt
52 kDa
41 kDa
WB : IκB
WB : Actin
41 kDa

## Slide 3
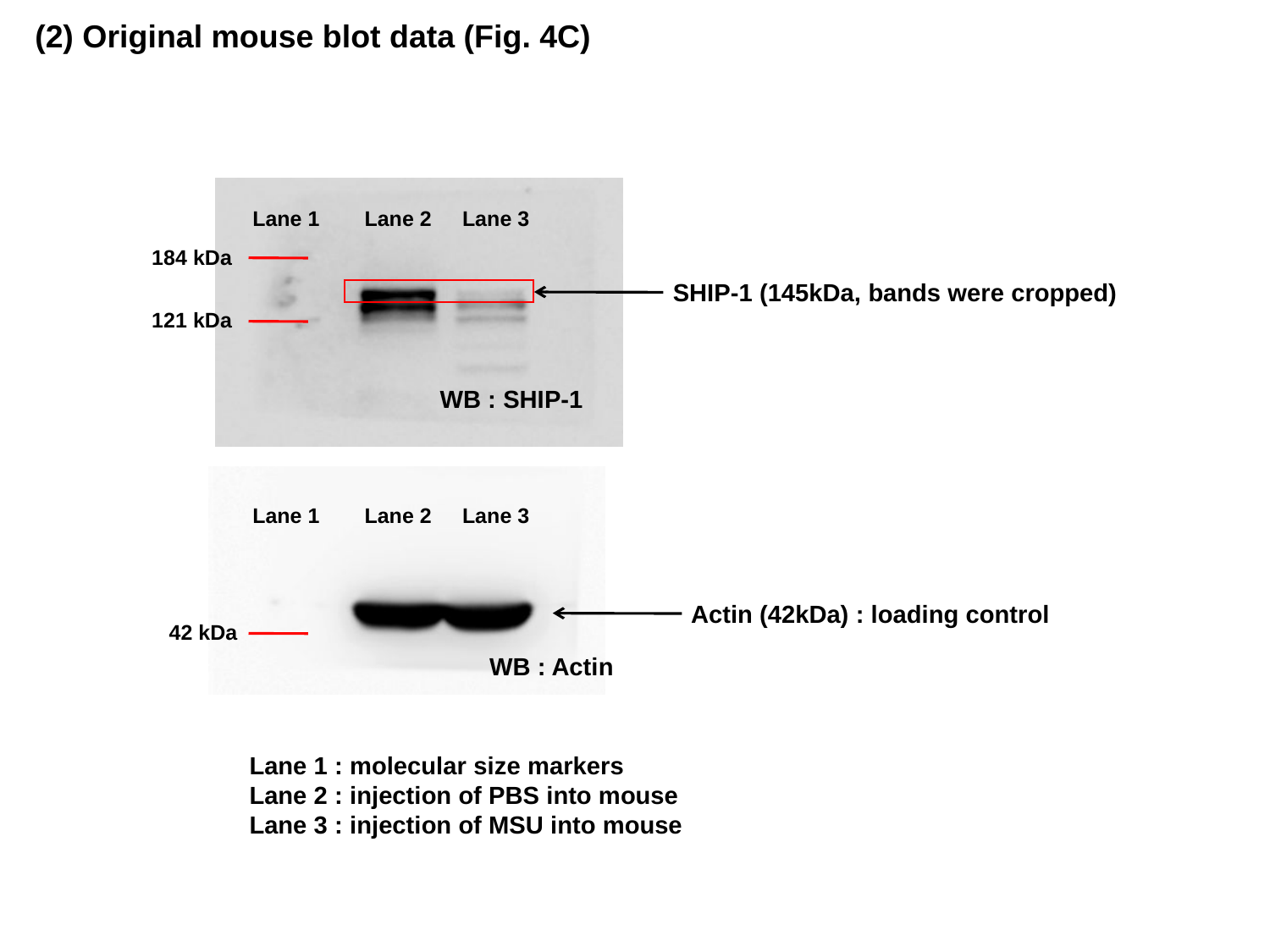

(2) Original mouse blot data (Fig. 4C)
Lane 1
Lane 2
Lane 3
184 kDa
SHIP-1 (145kDa, bands were cropped)
121 kDa
WB : SHIP-1
Lane 1
Lane 2
Lane 3
Actin (42kDa) : loading control
42 kDa
WB : Actin
Lane 1 : molecular size markers
Lane 2 : injection of PBS into mouse
Lane 3 : injection of MSU into mouse
